# A Reflectivity Enhanced 3D Optical Storage Nanostructure Application Based on Direct Laser Writing Lithography

**DOI:** 10.3390/ma16072668

**Published:** 2023-03-27

**Authors:** Lei Song, Dekun Yang, Zhidan Lei, Qimeng Sun, Zhiwen Chen, Yi Song

**Affiliations:** 1 The Institute of Technological Sciences, Wuhan University, Wuhan 430072, China; 2 School of Microelectronics, Wuhan University, Wuhan 430072, China

**Keywords:** optical storage, nanostructures, machine learning, direct laser writing lithography

## Abstract

To enable high-density optical storage, better storage media structures, diversified recording methods, and improved accuracy of readout schemes should be considered. In this study, we propose a novel three-dimensional (3D) sloppy nanostructure as the optical storage device, and this nanostructure can be fabricated using the 3D laser direct writing technology. It is a 900 nm high, 1 × 2 µm wide Si slope on a 200 nm SiO2 layer with 200 nm Si_3_N_4_ deposited on top to enhance reflectivity. In this study, we propose a reflected spectrum-based method as the readout recording strategy to stabilize information readout more stable. The corresponding reflected spectrum varied when the side wall angle of the slope and the azimuth angle of the nanostructure were tuned. In addition, an artificial neural network was applied to readout the stored information from the reflected spectrum. To simulate the realistic fabrication error and measurement error, a 20% noise level was added to the study. Our findings showed that the readout accuracy was 99.86% for all 120 data sequences when the slope and azimuth angle were varied. We investigated the possibility of a higher storage density to fully demonstrate the storage superiority of this designed structure. Our findings also showed that the readout accuracy can reach its highest level at 97.25% when the storage step of the encoded structure becomes 7.5 times smaller. The study provides the possibility to further explore different nanostructures to achieve high-density optical storage.

## 1. Introduction

With the advent of the information age, optical information storage is undergoing rapid development. In the past few years, compact disks, digital versatile disks, and Blu-ray disks (BD) [1,2,3,4] were the most widely used optical information storage devices. These storage devices have a long lifespan, relatively high storage density, and low cost [5,6,7]. However, they are associated with small capacities, low read/write and recording speeds, and a high native bit error rate because of which researchers are in search of a better storage method to replace optical disk storage. The information storage system is still a relatively weak and critical link in the development of information technology. Hence, the current storage density and data transmission rate achieved by optical storage are still far from meeting the requirements of the rapidly developing information sciences and technologies. Thus, optical storage is being improved from long-wave to short-wave, low-dimensional to high-dimensional (i.e., from flat to three-dimensional), far-field to near-field, photothermal effect to photonic effect, and point-by-point storage to parallel storage in order to enhance its storage and data transmission capability. In the past few years, several approaches have been proposed for increasing optical storage density. Some of these approaches include multi-wavelength and multi-level storage [8,9], fluorescent nanocrystals [10], two-photon excitation [11], optical near-field recording [12], polarization modulation [13], readout scheme-dependent (DNA-based [14]), and volume product-dependent (holographic memory [15]). However, each approach is associated with certain inevitable drawbacks. For example, it is difficult to find suitable storage media for multi-wavelength and multi-level storage, two-photon excitation, fluorescent nanocrystals, and optical near-field recording. Polarization modulation and readout scheme-dependent (DNA-based) signals require precise patterning and complex readout schemes. Volume product-dependent (holographic memory) is difficult to obtain for full product industrialization solutions [16]. Hence, more research into developing new storage methods has been carried out.

Photonic nanostructures have a wide scattering cross-section and can control light at the nanoscale level, highlighting the potential applications of nanostructures in the field of optical storage density. Significant changes in the reflectance or transmittance of the material between light and matter under the action of localized surface plasmons [17,18] and Mie resonance [19,20] can produce structural colors that cover the spectrum. However, optical responses generated by different nanostructures may be similar or even convergent, indicating the requirement for more precise response decoding methods. Therefore, scattering spectra are used to encode the information, while artificial neural networks (ANN) in machine learning, an auxiliary tool, are used to decode the information [21,22,23,24]. The application of ANN to optical storage density allows it to identify and process spectra more accurately.

Moreover, the application of ANN can be extended to the field of optical nanostructure storage [25]. Although ANN is an effective tool, some of its structures generate an optical response that is extremely difficult to solve, thus affecting the density of optical storage. Weicha et al. decoded scattered spectra beyond the diffraction limit by using a deep learning algorithm. They achieved an accuracy of 95.84% by adjusting the noise in the training sample to 20% [26]. Other studies have reported the effectiveness of ANN-based auxiliary methods in coding optical information.

In this study, we propose a novel three-dimensional (3D) sloppy nanostructure as an optical storage device. The bottom surface of the sloppy structure forms a non-perpendicular angle (slope angle) with the sides. By changing the slope angle as well as the azimuth of the structure, different optical signals can be obtained. A specific combination of two angles represents a coding structure, this strategy can enhance the storage density under the condition of fixed spatial resources. We can obtain different reflectance spectra based on the variation of the slope angle and the azimuth angle of the nanostructure. Accordingly, we propose wavelength-based reflectance spectra as a readout recording strategy to stabilize the information readout given the high sensitivity of the spectra.

In this paper, the optical spectra obtained by changing the slope angle are used as the input to the ANN, and the encoded data sequence is used as the output of the ANN. Due to the excellent image recognition function of ANN, the mapping between data sequences and the spectra can be trained. A spectrum corresponds to a structure encoded by one data, so it can be used as a medium for encoding and decoding.

We added 20% noise to the ANN training set samples to simulate realistic manufacturing and measurement errors. The readout accuracy was found to be 99.86% for all 120 data sequences. We reduced the azimuthal variation interval of the slope angle and the whole nanostructure to achieve higher-density optical storage and found that the readout accuracy can still reach 97.25%.

## 2. Optical Data Information Encoding

### 2.1. Data Encoding Strategy

Direct laser writing lithography (DLWL) [27,28] is a widely used technique to fabricate a 3D structure. It is a maskless lithography technique that can customize arbitrary 3D structure on a photoresist with sub-micron accuracy. A real 3D device can be formed with selected materials and structures, following etching, deposition, and other processes. Compared to the disadvantages of the digital micro-mirror device (DMD) with surface roughness over 1 μm [29] and the femtosecond-laser-induced two-photon polymerization (TPP) with low efficiency for large area manufacturing [30], DLWL device used in our lab can achieve 300 nm resolution with the surface roughness below 6 nm, which means guaranteed optical properties. In addition, the DLWL technology can reach 4096 exposure grayscale, i.e., a high resolution of 12-bit grayscale level, which can make it achieve high writing resolution in the vertical direction. Compared with other methods, large-area microstructures can be transferred to other materials without the need for complex molds [31]. Only one mold needs to be made for mass production.

**Figure 1:**
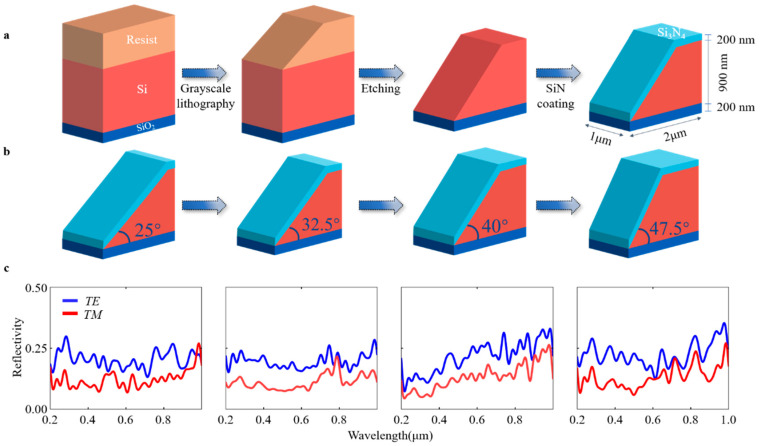
The flow chart of the manufacturing process and encoding strategy. (**a**) The nanostructure manufacturing process, (**b**) Variation in structure according to slope angle, and (**c**) Wavelength-based reflectivity spectra of different slope angles.

In this condition, we propose a 3D optical storage nanostructure scheme based on a DLWL device. In the experiments, four samples with different slope angles were produced using a laser direct writing device (PICOMASTER 100, 4PICO, Eindhoven, The Netherlands). The surface smoothness measurements using a 3D profilometer are shown in Figures S1 and S2 of the Supplementary Materials. The scheme uses different reflection spectral responses from different patterns to encode information. The structure and materials, used as inputs to the numerical model are shown in Figure 1a. As a thin film, the Si_3_N_4_ layer, which has a height of 200 nm, was deposited on a silicon-on-insulator (SOI) substrate, which has a 900 nm silicon overlay. The SOI substrate has excellent stability, oxidation, and aging resistance [32]. This size was chosen according to the etching ability of grayscale lithography. The Z-axis height was precisely controlled in the 3D laser lithography process, and the selection of SOI substrate at this height allowed for better fine tuning of the slope angle. Depositing a Si_3_N_4_ film of 200 nm thickness can largely increase the reflectivity of the structure due to the high reflectivity of the Si_3_N_4_ film, thus improving the accuracy of the readout and enabling a high density of optical storage. In order to simulate the reflective spectra, a rigorous coupled-wave analysis method was applied. In this study, we used the DiffractMOD module of the simulation software R-soft, setting the harmonics in the module to 15.

The simulated results of different structures with different slope angles (Figure 1b) revealed different wavelength-based reflectance spectra, with spectra processed by a 3 × 3 high-pass filter (Figure 1c). In this study, light in the wavelength range of 200 nm to 1000 nm was chosen in order to simulate the real experimental setting of the readout system. In the spectra, the blue and red lines represent Transverse Electric *(TE)/*Transverse Magnetic *(TM)* polarization, respectively. The spectral responses of *TE* and *TM* were significantly different, suggesting that more information can be collected with both *TE* and *TM* polarizations. The spectral responses can be easily distinguished for different structures, suggesting the potential applications of special structures with different slope angles in optical storage encoding.

**Figure 2:**
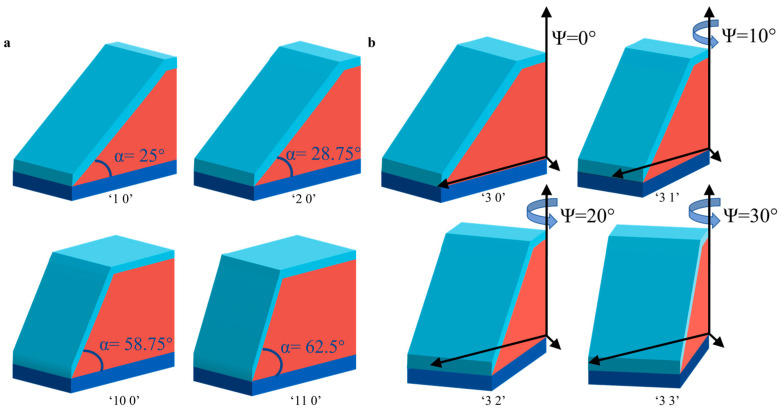
Description of the encoding method: The “α ψ” encoding form is used, with α indicating the slope angle and ψ indicating the azimuth of the overall structure; “1 0” indicates data information encoded for a structure with a slope angle of 25° and an azimuth of 0°. (**a**) for each 3.75° increase in slope angle, α increases by one; (**b**) for each 10° increase in azimuth angle, ψ increases by one.

We designed two different storage strategies, i.e., slope angle-based and azimuth angle-based, to use the capability and flexibility of 3D laser direct writing. The slope angles of nanostructures vary from 25° to 66.25°, with the interval between two adjacent structures being 3.75° (shown in Figure 2a). The choice of slope angle is based on the size of the width and height of Si to ensure a flat top that is feasible for fabrication. With respect to defining the 12 structures, the structure with the slope angle of 25° and the azimuth angle of 0° is encoded as “1 0.” As the slope angle increases to 66.25°, the coding number increases up to “12 0”. As shown in Figure 2b, the nanostructures have a slope angle of 32.5°, and the azimuth angle changes from 0° to 180°, with a 10° interval between two adjacent structures. With respect to defining the 10 structures, the structure with the slope angle of 32.5° and the azimuth angle of 0° is encoded as “3 0”. As the slope angle increases to 180°, the coding number increases to “3 18”.

### 2.2. Data Encoding Based on Structure-Specific Slope Angle

**Figure 3:**
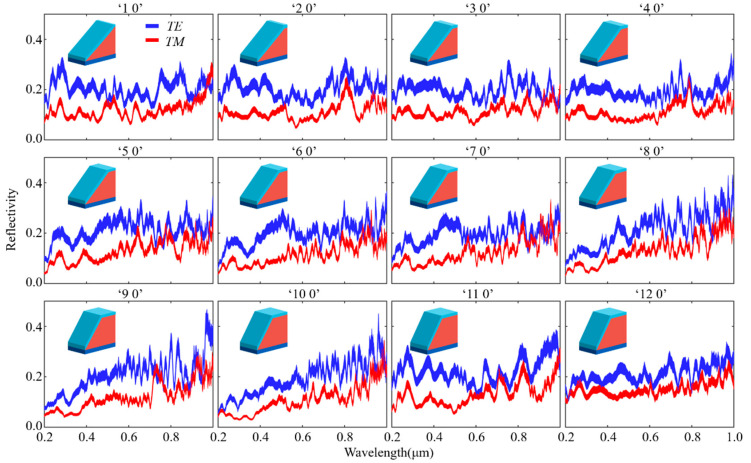
Simulated wavelength-dependent spectra for 12 data sequences (Azimuth fixed to 0°).

Figure 3 shows the spectrum under *TE/TM* polarizations for structures having increasing slope angles. Twelve reflectance spectra were obtained when the azimuth angle of the structure was fixed at 0° and the slope angle was adjusted from 25° to 66.25° at an interval of 3.75°. 

### 2.3. Data Encoding Based on Azimuthal Variation

**Figure 4:**
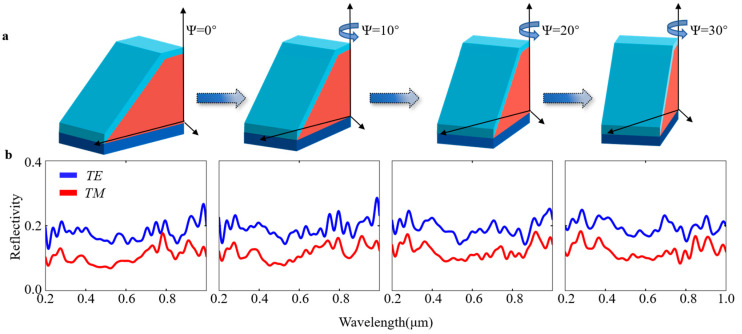
The encoding strategy. (**a**) Variation in structure according to azimuth, (**b**) wavelength-dependent spectra of different azimuths.

Currently, 3D laser lithography enables the custom processing of almost arbitrary structures. Thus, using the change in azimuth angles of the structures (i.e., different orientations of the structures) is a reliable method to achieve optical storage encoding (Figure 4a). The simulating results of different structures with different azimuth angles revealed different wavelength-based reflectance spectra, with spectra processed by a 3 × 3 high-pass filter (Figure 4b). 

**Figure 5:**
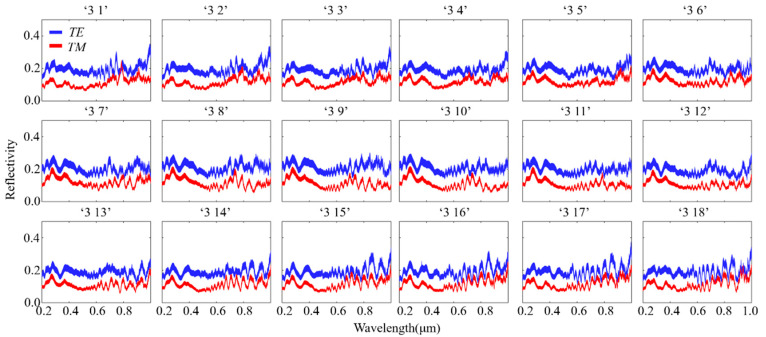
Simulated wavelength-dependent spectra for 18 data sequences (Fixed slope angle).

Figure 5 shows the reflectance spectra under *TE*/*TM* polarizations for the structures when the azimuth angle is increased. Eighteen different spectral responses were obtained from different structures.

### 2.4. Data Encoding Combined Structure-Specific Slope Angle and Azimuthal Variation

**Figure 6:**
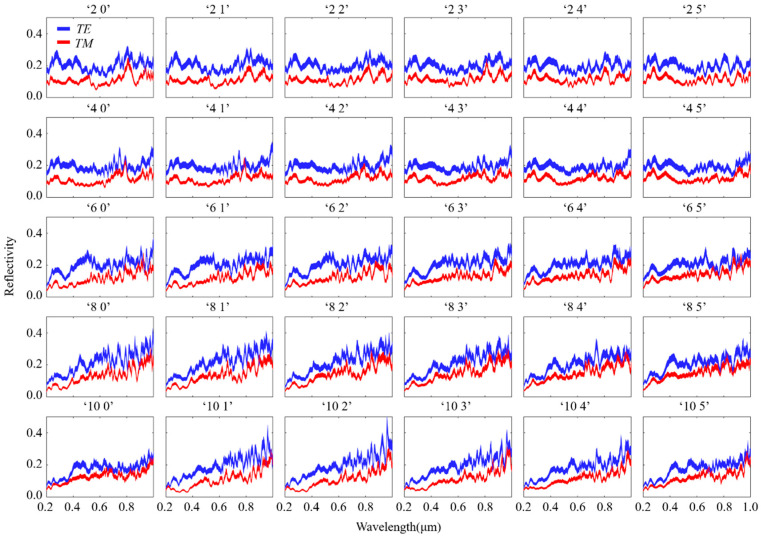
Simulated wavelength-dependent spectra for 30 of the 120 data sequences (slope angle combined with azimuth).

The slope angle of the structure was combined with the azimuth angle to improve storage density and encoded as “α ψ,” where α represents the slope angle and ψ represents the azimuth angle of the structure. We chose a total of 12 slope angles from 25° to 66.25° and 10 azimuth angles from 0° to 90°. Figure 6 shows the 30 spectral responses of the 120 data. The codes “ 2 0 “ up to “ 2 5 “ indicate a structure with a fixed slope angle of 28.75° and an azimuth angle increasing from 0 to 50°. The codes “4 0” and “6 0” indicate a structure with a fixed azimuth angle of 0° and slope angles of 36.25° and 43.75°, respectively. Our findings showed that the changes in spectral response increased with the changes in slope angle and azimuth angle. This finding is beneficial for subsequent decoding work.

## 3. Analysis and Evaluation of Readout Information

### 3.1. Optical Data Information Readout Based on ANN

**Figure 7:**
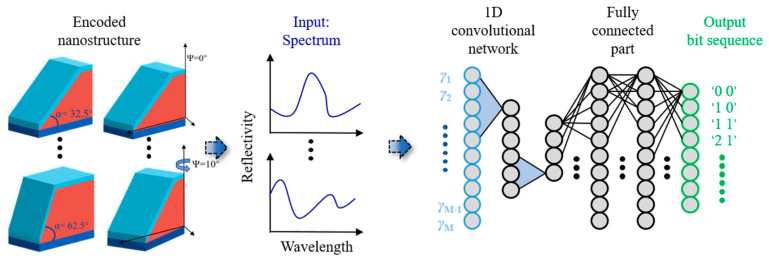
Schematic diagram of a convolutional ANN. The spectra corresponding to the different sloppy structures are used as inputs to ANN; the encoded data information is used as the ANN output.

In this study, machine learning was used to decode the data and read the dataset. The convolutional neural network (CNN) used in this study is a type of ANN (as shown in Figure 7), which can effectively solve the pattern recognition problem and facilitate matching network connections. The CNN with a “leaky ReLU” function is categorized into three levels. The number of filters in CNNs’ three channels with kernel sizes of 7, 5, and 3 was 32, 32, and 1, respectively. Each layer of the CNN was also equally normalized. It also connected the maximum ensemble layer with a kernel size of two. There were two layers of a fully connected network, with 64 and 32 neurons in each layer that were activated using “tanh.” A spectrum consisting of *TE* or *TM* polarization was introduced as a parallel channel in the input layer of the ANN with a kernel size of seven for each channel.

In this study, we introduced *TE*- or *TM*-polarized spectra generated from different sloppy structures as parallel channels into the input level of the ANN during its training process and fed their corresponding encoded data into the output level. A total of 300 wavelength-based spectra were generated using the simulator R-soft, which was used to train the ANN. The number of training and testing spectrums is set in a ratio of 7:3. In the readout program, we sent the spectra to the trained ANN and chose appropriate networks to propagate forward so as to obtain the encoded data on the retrieval structure. In the subsequent steps, the training quality was evaluated using cross-entropy loss and validation accuracy.

### 3.2. Readout Result Based on Spectrum Response

**Figure 8:**
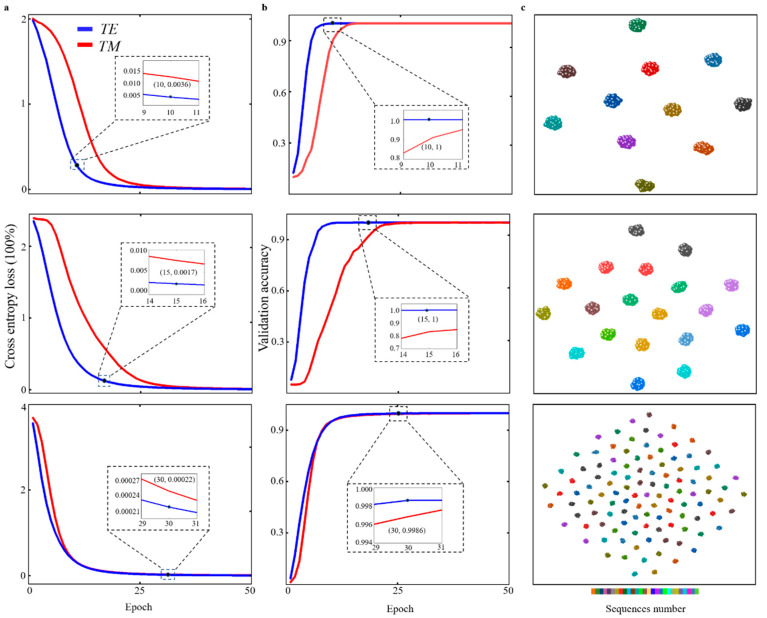
Demonstration of training quality and readout accuracy. (**a**) Cross-entropy loss for 12, 18, and 120 data sequences trained using *TE* polarization and *TM* polarization, (**b**) Validation accuracy for 12, 18, and 120 data sequences trained using *TE* polarization and *TM* polarization, (**c**) 12, 18, and 120 data sets of *TE* polarized training sets with a t-SNE spot.

To prepare an appropriate AAN training sequence, 300 spectra were randomly generated for 120 structures, when the wavelength was between 200 nm and 1000 nm, within a 20% noise level of the initial spectrum [33]. Out of the 20% spectral noise considered in this research, 10% was used to simulate errors due to manufacturing, and the remaining 10% was used to simulate noise based on measurement. Both manufacturing error and measurement error have uncontrollable factors that will affect the actual readout accuracy. The manufacturing noise and measurement noise vary according to the actual measurement equipment and environment. According to the research of D. Yang et al. [16], it is reasonable to set the manufacturing noise and measurement noise at 10%, respectively. Figure 8a shows the cross-entropy loss corresponding to the 12, 18, and 120 data sets at different polarizations. Figure 8b shows the validation accuracy corresponding to the 12, 18, and 120 data sets at different polarizations. With the increase in epoch, the training using *TE* polarization converged faster than that using *TM* polarization. As shown in Figure 8a,b, in the training of 12 data sets using *TE* polarization, the validation accuracy at the 10th epoch was 100%, with a corresponding loss of 0.0036. Similarly, in the training of 18 data sets using *TE* polarization, the validation accuracy was 100% with a relative loss of 0.0017. The validation accuracy reaches 99.86% in the epoch, with a loss of 0.00022 in training with 120 data sequences. It can be clearly seen that the convergence rate gradually slows down as the number of data sets increases, but the verification accuracy undergoes only minor changes. This may be due to the appropriate selection of steps in our encoding strategy. If the step chosen is too small, the verification accuracy may be greatly reduced. For example, in the data encoding pattern “α ψ” in this study, the step of α and ψ is set to 3.75° and 10°, respectively, which ensures enough encoded data sequences without affecting the quality of training. In the case that more coding data sequences are needed in future work, a smaller step can be fixed. 

The t-distributed stochastic neighbor embedding (t-SNE) was used to assess the uniqueness of different structures and analyze data sets [34]. In the t-SNE scatter plot, well-distributed scattered spots represented identifiable structures based on data sets, but neighboring and overlapping spots implied that the data had some similarity. Figure 8c shows the results for 12, 18, and 120 data sets at *TE* and *TM* polarization. As shown in the figure, the error was set to 20%. Different colors are matched to different structures, while each spot represents a simulated spectrum. In the figure of t-SNE, the data sets of each structure are well distinguished, which explains the ability of ANN to correctly recognize information on data with 100% accuracy. 

## 4. Toward Higher Storage Density

**Figure 9:**
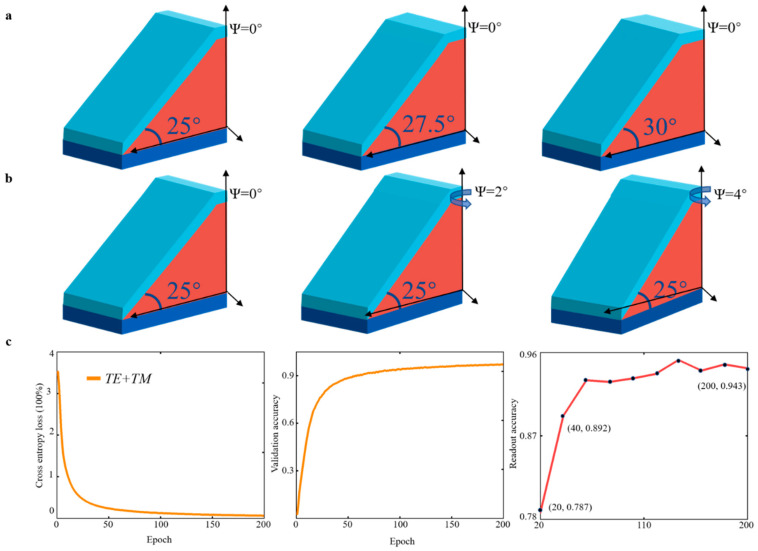
Description of the encoding method and readout accuracy: (**a**) The azimuth angle ψ is fixed (0°), and the slope angle α increases gradually in 2.5° intervals from 25° to 62.5°. (**b**) The slope angle α is fixed (25°), and the azimuth angle ψ increases gradually from 0° to 10° at 2° intervals (**c**) *TE + TM* polarization for cross-entropy loss and training for validation accuracy; 96 data sets of reduced wavelength spectra for network testing for readout accuracy.

The interval between each structure was reduced, and the original encoding (shown in Figure 2) was kept unchanged to achieve high-density storage of this particular structure. As shown in Figure 9a,b, we changed the interval of α from 3.75° to 2.5° and the interval of ψ from 10° to 2° in “α ψ.” In addition, α was increased from 25° to 66.25°, and ψ was increased from 0° to 10°. In this study, we reduced the number of overall samples to investigate the possibility of improving storage accuracy under the circumstances of smaller steps. The “α ψ” is coded as “1 0,” which means that the slope angle is 25° and the azimuth angle is 0°. If the slope angle is 27.5° and the azimuth angle is 4°, the “α ψ” is coded as “2 2.” We considered a different approach from the original one and used the dual channel mode *TE* + *TM* to read out the encoded data more accurately. This approach was used because the step of the structure corresponding to the encoded data becomes smaller than the original one, and the dual channel can read it out better. Figure 9c shows the readout accuracy of the test set, and the accuracy of the training set readout reached 97.25% at 200 epochs. We set the test for every 20 epochs and found that the test set readout accuracy reached 94.3% at 200 epochs, suggesting that the proposed storage strategy can achieve the data-information storage. In this condition, per 2 × 1 × 1.3 microns can reach 6480 (36 × 180) data-information storage, approximately 2^13^ bit per 2 × 1 × 1.3 microns.

## 5. Conclusions

In the present research, a scheme for encoding data for specific nanostructures is presented. This present research was based on the different reflection spectra of different structures in 3D laser lithography. The data information encoding method for 3D nanostructure recording has greater advantages compared with 2D nanostructure recording methods. In this study, we used deep learning to identify the data encoded by each structure and enable high-density optical storage and highly accurate storage of data information.

We also proposed a recording method that combines wavelength-dependent spectroscopy to identify the data information encoded by that structure using ANN. We found that the readout accuracy of 12, 18, and 120 sequences can reach 100% after 10, 15, and 30 training sessions. Meanwhile, the good t-SNE figure obtained by separation can be well evaluated for the uniqueness of this particular structure.

This particular structure is not symmetric compared with many optical storage structures. Thus, we do not need to worry about the similarity between *TE* and *TM* polarization spectra due to the 0° and 180° angles of the overall structure azimuth.

We also reduced the size of the encoded structure in order to evaluate the limit of achieving high-density optical storage for each structure. Our findings showed that the overall readout accuracy can be as high as 97.25% when the slope angle represented by α in the encoding method is changed from 3.75° to a 2.5° interval and the azimuth angle of the structure represented by ψ was changed from 10° to a 2° interval. This accuracy level is sufficient to demonstrate the effectiveness of this specific nanostructure in achieving high-density optical storage. If the processing error and system error are further reduced and the ANN algorithm is further improved to increase readout accuracy, smaller steps can be distinguished, thus achieving higher storage density.

The proposed 3D optical storage nanostructure in this study ensures sufficient encoding sequences and improves the training quality according to the change in the slope angle and azimuth of the structure. It can theoretically achieve a high storage density of 6480 (36 × 180) data-information storage per 2 × 1 × 1.3 μm, approximately 2^13^ bit per 2 × 1 × 1.3 microns, which provides guidance for high-density optical storage research.

## 6. Prospection

As the precision of laser direct writing equipment continues to improve, 3D lithography can precisely adjust the device size, the step of slope angle between different structures can be further reduced to 1° and the step of azimuth angle can be reduced to 1°, so the arrangement combination of all structures is 3.24 × 10^4^ (90 × 360). In this condition, per 2 × 1 × 1.3 microns can reach 3.24 × 10^4^ (90 × 360) data information storage, i.e., 2^15^ bit.

## Data Availability

The data presented in this study are available on request from the corresponding author.

## Supplementary Materials

The following supporting information can be downloaded at: https://www.mdpi.com/article/10.3390/ma16072668/s1, Figure S1: Image of 3D lithographic structure taken with 3D optical profilometer; Figure S2: Image of 3D lithographic structure taken with 3D optical profilometer.

## References

[1] Satoh I., Ohara S., Akahira N., Takenaga M. (1998). Key technology for high density rewritable DVD (DVD-RAM). IEEE Trans. Magn..

[2] Borg H.J., Van Schijndel M., Rijpers J.C.N., Lankhorst M.H.R., Zhou G., Dekker M.J., Ubbens I.P.D., Kuijper M. (2001). Phase-change media for high-numerical-aperture and blue-wavelength recording. Jpn. J. Appl. Phys..

[3] Taylor A.B., Kim J., Chon J.W.M. (2012). Detuned surface plasmon resonance scattering of gold nanorods for continuous wave multilayered optical recording and readout. Opt. Express.

[4] Taylor A.B., Michaux P., Mohsin A.S.M., Chon J.W.M. (2014). Electron-beam lithography of plasmonic nanorod arrays for multilayered optical storage. Opt. Express.

[5] Zhang J., Gecevičius M., Beresna M., Kazansky P.G. (2014). Seemingly unlimited lifetime data storage in nanostructured glass. Phys. Rev. Lett..

[6] Gu M., Li X., Cao Y. (2014). Optical storage arrays: A perspective for future big data storage. Light Sci. Appl..

[7] Gu M., Zhang Q., Lamon S. (2016). Nanomaterials for optical data storage. Nat. Rev. Mater..

[8] Xu D., Hu H., He L. Multi-wavelength and multi-level optical storage based on photochromic materials. Proceedings of the Seventh International Symposium on Optical Storage (ISOS 2005).

[9] Xu D. (2016). Multi-Dimensional Optical Storage.

[10] Riesen N., Pan X., Badek K., Ruan Y., Monro T.M., Zhao J., Ebendorff-Heidepriem H., Riesen H. (2018). Towards rewritable multilevel optical data storage in single nanocrystals. Opt. Express.

[11] Liu T.C., Zhang L., Sun J. (2018). Optical properties of dithienylethene and its applications in super-resolution optical storage. Chin. J. Lasers.

[12] Tominaga J., Nakano T., Atoda N. (1998). An approach for recording and readout beyond the diffraction limit with an Sb thin film. Appl. Phys. Lett..

[13] Zeng B.J., Ni R.W., Huang J.Z., Li Z., Miao X.S. (2014). Polarization-based multiple-bit optical data storage. J. Opt..

[14] Mottaghi M.D., Dwyer C. (2013). Thousand-Fold Increase in Optical Storage Density by Polychromatic Address Multiplexing on Self-Assembled DNA Nanostructures. Adv. Mater..

[15] Coufal H.J., Psaltis D., Sincerbox G.T. (2000). Holographic Data Storage.

[16] Yang D., Lei Z., Li L., Shen W., Li H., Gui C., Song Y. (2023). High optical storage density using three-dimensional hybrid nanostructures based on machine learning. Opt. Lasers Eng..

[17] Lee H.-E., Kim R.M., Ahn H.-Y., Lee Y.Y., Byun G.H., Im S.W., Mun J., Rho J., Nam K.T. (2020). Cysteine-encoded chirality evolution in plasmonic rhombic dodecahedral gold nanoparticles. Nat. Commun..

[18] Kuzyk A., Schreiber R., Fan Z., Pardatscher G., Roller E.-M., Högele A., Simmel F.C., Govorov A.O., Liedl T. (2012). DNA-based self-assembly of chiral plasmonic nanostructures with tailored optical response. Nature.

[19] Kuznetsov A.I., Miroshnichenko A.E., Brongersma M.L., Kivshar Y.S., Luk’yanchuk B. (2016). Optically resonant dielectric nanostructures. Science.

[20] Park C.-S., Shrestha V.R., Yue W., Gao S., Lee S.-S., Kim E.-S., Choi D.-Y. (2017). Structural color filters enabled by a dielectric metasurface incorporating hydrogenated amorphous silicon nanodisks. Sci. Rep..

[21] Fang J., Swain A., Unni R., Zheng Y. (2021). Decoding optical data with machine learning. Laser Photon. Rev..

[22] Li J., Zhang M., Wang D. (2017). Adaptive demodulator using machine learning for orbital angular momentum shift keying. IEEE Photonics Technol. Lett..

[23] Doster T., Watnik A.T. (2017). Machine learning approach to OAM beam demultiplexing via convolutional neural networks. Appl. Opt..

[24] Chugh S., Gulistan A., Ghosh S., Rahman B.M.A. (2019). Machine learning approach for computing optical properties of a photonic crystal fiber. Opt. Express.

[25] Abiodun O.I., Jantan A., Omolara A.E., Dada K.V., Mohamed N.A., Arshad H. (2018). State-of-the-art in artificial neural network applications: A survey. Heliyon.

[26] Wiecha P.R., Lecestre A., Mallet N., Larrieu G. (2019). Pushing the limits of optical information storage using deep learning. Nat. Nanotechnol..

[27] Luo S., Hoff B.H., Maier S.A., de Mello J.C. (2021). Scalable Fabrication of Metallic Nanogaps at the Sub-10 nm Level. Adv. Sci..

[28] Bernardeschi I., Ilyas M., Beccai L. (2021). A review on active 3D microstructures via direct laser lithography. Adv. Intell. Syst..

[29] Sun C., Fang N., Wu D.M., Zhang X. (2005). Projection micro-stereolithography using digital micro-mirror dynamic mask. Sensors Actuators A Phys..

[30] Guo R., Xiao S., Zhai X., Li J., Xia A., Huang W. (2006). Micro lens fabrication by means of femtosecond two photon photopolymerization. Opt. Express.

[31] Li W., Yu M., Sun J., Mochizuki K., Chen S., Zheng H., Li J., Yao S., Wu H., Ong B.S. (2019). Crack engineering for the construction of arbitrary hierarchical architectures. Proc. Natl. Acad. Sci. USA.

[32] Rudenko T.E., Nazarov A.N., Lysenko V.S. (2020). The advancement of silicon-on-insulator (SOI) devices and their basic properties. Semicond. Phys. Quantum Electron. Optoelectron..

[33] Wiecha P.R., Arbouet A., Girard C., Lecestre A., Larrieu G., Paillard V. (2017). Evolutionary multi-objective optimization of colour pixels based on dielectric nanoantennas. Nat. Nanotechnol..

[34] Van der Maaten L., Hinton G. (2008). Visualizing data using t-SNE. J. Mach. Learn. Res..

