# Supplementary material for: A Reflectivity Enhanced 3D Optical Storage Nanostructure Application Based on Direct Laser Writing Lithography
Source: Materials (Basel). 2023 Mar 27;16(7):2668. doi: 10.3390/ma16072668 (PMC10095977; doi:10.3390/ma16072668)
Supplement: Supplementary file 1 [file materials-16-02668-s001.zip › materials-2274062-supplementary.pdf]

materials

MDPI

Supplementary

A Reflectivity Enhanced 3D Optical Storage Nanostructure Application Based on Direct Laser Writing Lithography

Lei Song [1,†], Dekun Yang [1,†], Zhidan Lei [1,†], Qimeng Sun [1], Zhiwen Chen [1,*] and Yi Song [1,2,*]

Sample images with different slope angle fabricated by the laser direct writing device

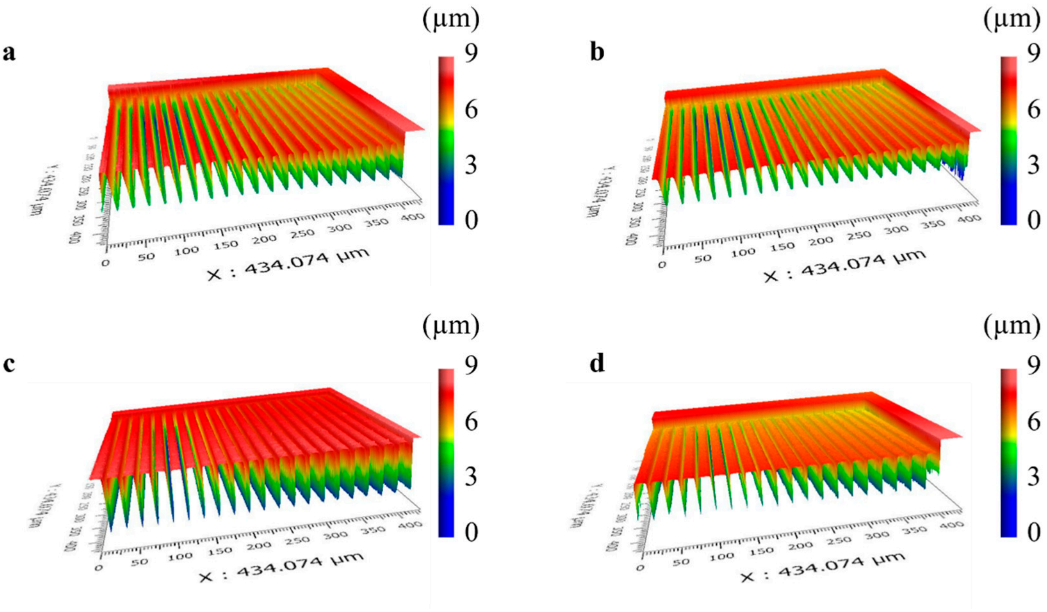

Figure S1. Image of 3D lithographic structure taken with 3D optical profilometer. **a** Sample with 60° slope angle structure, **b** Sample with 62.5° slope angle structure, **c** Sample with 65° slope angle structure, **d** Sample with 67.5° slope angle structure.

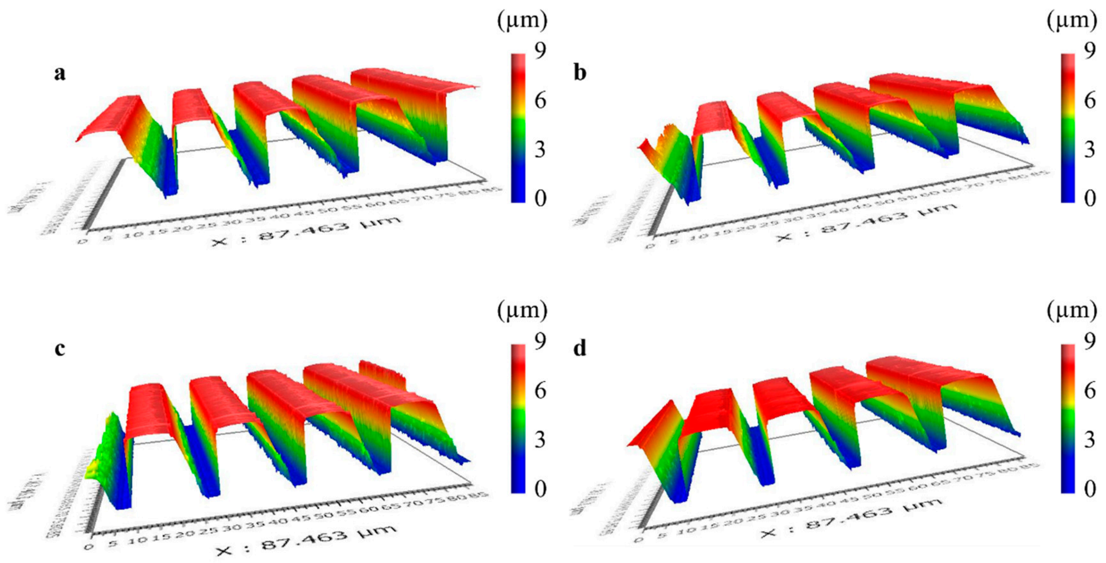

Figure S2. Image of 3D lithographic structure taken with 3D optical profilometer. **a** Sample with 60° slope angle structure, **b** Sample with 62.5° slope angle structure, **c** Sample with 65° slope angle structure, **d** Sample with 67.5° slope angle structure.
